# Adjuvant therapeutic strategy decision support for an elderly population with localized breast cancer: A monocentric cohort retrospective study

**DOI:** 10.1371/journal.pone.0290566

**Published:** 2023-08-24

**Authors:** Julia L. Fleck, Daniëlle Hooijenga, Raksmey Phan, Xiaolan Xie, Vincent Augusto, Pierre-Etienne Heudel

**Affiliations:** 1 Mines Saint-Etienne, Univ Clermont Auvergne, CNRS, UMR 6158 LIMOS, Centre CIS, Saint-Etienne, France; 2 Medical Oncology Department, Centre Léon Bérard, Lyon, France; University of Vermont College of Medicine: University of Vermont Larner College of Medicine, UNITED STATES

## Abstract

Guidelines for the management of elderly patients with early breast cancer are scarce. Additional adjuvant systemic treatment to surgery for early breast cancer in elderly populations is challenged by increasing comorbidities with age. In non-metastatic settings, treatment decisions are often made under considerable uncertainty; this commonly leads to undertreatment and, consequently, poorer outcomes. This study aimed to develop a decision support tool that can help to identify candidate adjuvant post-surgery treatment schemes for elderly breast cancer patients based on tumor and patient characteristics. Our approach was to generate predictions of patient outcomes for different courses of action; these predictions can, in turn, be used to inform clinical decisions for new patients. We used a cohort of elderly patients (≥ 70 years) who underwent surgery with curative intent for early breast cancer to train the models. We tested seven classification algorithms using 5-fold cross-validation, with 80% of the data being randomly selected for training and the remaining 20% for testing. We assessed model performance using accuracy, precision, recall, F1-score, and AUC score. We used an autoencoder to perform dimensionality reduction prior to classification. We observed consistently better performance using logistic regression and linear discriminant analysis models when compared to the other models we tested. Classification performance generally improved when an autoencoder was used, except for when we predicted the need for adjuvant treatment. We obtained overall best results using a logistic regression model without autoencoding to predict the need for adjuvant treatment (F1-score = 0.869).

## Introduction

Age is an established risk factor for breast cancer. The age threshold that typically characterizes elderly patients in high income countries is 65 years. In the United States, the median age of diagnosis of breast cancer for women is 62 [1], and 30% of new breast cancer cases in 2020 were diagnosed in women aged 70 years or more [2]. In European Union countries (EU-27), approximately 44% of breast cancer cases occur in women older than 65 years of age [3]. Guidelines for the management of elderly patients with early breast cancer are scarce, primarily due to the lack of evidence, including the lack of validation of online adjuvant therapy tools. As a result, in non-metastatic settings, treatment decisions are often made under considerable uncertainty; this commonly leads to undertreatment and, consequently, poorer outcomes [4, 5].

Treatment plans for breast cancer vary depending on the type of breast cancer, its stage, as well as other factors such as patient preferences and overall health. In early breast cancer, current standard protocols typically consist of surgery accompanied by either radiation therapy, neoadjuvant or adjuvant systemic therapy, or a combination of these therapies. The choice of post-surgery treatment for elderly breast cancer patients is generally considered a difficult decision because these patients are often in a worse physiological state. Elderly patients are rarely included in randomized clinical trials and underrepresented in meta-analyses showing a benefit of adjuvant chemotherapy with regard to breast-cancer mortality and overall survival chemotherapy [6, 7]. In the absence of clinical trial results, an alternative approach is to use artificial intelligence (AI) to assist with treatment decision making.

Some of the earliest AI applications to provide cancer treatment recommendations were knowledge-based systems [8, 9]. More recently, a variety of machine-learning approaches have been proposed to assist clinicians and/or breast cancer patients [10–15]. However, recommendations from existing decision support tools are usually relevant for patients aged 18 to 65 years, which is the age range for which most of the advisory tools have been trained. Relatively few studies analyzed treatment outcomes for elderly breast cancer patients [16–19].

One of the major prognostic tools in current clinical use for breast cancer is PREDICT (https://predict.nhs.uk) [20]. In spite of its popularity, PREDICT has been shown to under-perform in specific subgroups of patients, in particular older patients [21]. The recently developed Adjutorium (https://vanderschaar-lab.com/adjutorium/) is a breast cancer prognostication and treatment benefit prediction model that outperforms PREDICT [19]. Adjutorium used large-scale publicly available datasets from the United Kingdom and the United States consisting primarily of patients aged 30–65 years, along with a smaller subset of older patients (age > 65 at diagnosis). Due to limitations of the datasets, Adjutorium did not include important tumor information such as progesterone receptor (PR) status. In this study, we generate models that address both these limitations. For one, our cohorts are highly representative of elderly breast cancer patients because they include only patients aged 70 years or more. In addition, we make use of an extensive dataset that includes administrative, biological, treatment, primary tumor, and survival data.

Here we present a data-driven prediction tool that can provide recommendations for post-surgery treatment for elderly breast cancer patients. Using data from a cohort of elderly women (≥ 70 years) diagnosed with cancer who underwent surgery with curative intent for early breast cancer, we predict all-cause mortality at 5 years in four clinically relevant scenarios. Using our models, it is possible to compare expected outcomes (e.g., difference in patient survival) for different treatment options, and thus generate an integrated view of what will likely happen to the patient in different treatment scenarios. This information can help oncologists to identify candidate adjuvant treatment schemes for elderly breast cancer patients based on tumor and patient characteristics.

## Methods

### Recruitment

This retrospective study used individual pseudonymized data collected from all consecutive elderly women (≥ 70 years) diagnosed with cancer who underwent surgery with curative intent (lumpectomy or mastectomy +/- axillary lymph node dissection) for early breast cancer in the French comprehensive Léon Bérard Cancer Center, from January 1997 to December 2016. There were no restrictions considering breast cancer histological and molecular subtype, tumor size (from pT1 to pT4) or lymph node status (from pN0 to pN3). Patients were excluded in case of in situ carcinoma without infiltrative carcinoma and in case of distant metastasis at the time of breast surgery. Because we were interested in 5-year survival among elderly patients, in this study we only included patients who were followed for at least five years, and for whom information on vital status was available. A total of 976 patients met these inclusion criteria.

We used software ConSore to build our database. ConSore is a data mining tool developed by UNICANCER, a French academic cancer research organization [22]. Natural Language Processing is used to select patient cohorts and extract data from electronic medical records (EMR), providing a homogenous collection of meaningful information. Information is extracted in a structured form according to research criteria. It should be noted that a second human check was nevertheless carried out on all EMR studied.

Data collected in our analysis included the following patient characteristics at early breast cancer diagnosis: age, Eastern Cooperative Oncology Group performance status, body mass index (BMI), comorbidities (diabetes, cardiac insufficiency, coronary insufficiency, chronic obstructive pulmonary disease (COPD) and cognitive disorders), hospitalization history in the previous year, polypharmacy (> or = 5 medications a day). The following biologic measures at diagnosis were collected: hemoglobin, lymphocytes count, albuminemia, creatinine clearance (Cockcroft-Gault). The following data about disease characteristics were extracted: histological subtype, hormone receptor status, Human Epidermal Growth Factor Receptor-2 (HER2) status, Scarff-Bloom and Richardson (SBR) grade, number of tumors, size of the biggest tumor, and lymph node involvement according to the TNM classification [23]. The expression of estrogen receptors (ER), progesterone receptors (PR), and HER2 status were issued from the histopathological results of pre-therapeutic biopsy. Hormone receptor negativity was defined if less than 10% of cells stained for estrogen and progesterone receptors. The expression of HER2 was considered negative in case of lower than 1+ immunohistochemistry staining. For tumors with a score of 2+, an additional in situ hybridization determined HER2 amplification or non-amplification [24]. Data about cancer treatments included: type of surgery, lymph node dissection, adjuvant radiotherapy, adjuvant chemotherapy, adjuvant HER2 targeted therapy, adjuvant endocrine therapy. Continuous variables were categorized based on expert opinion while categorical variables with more than two categories were dichotomized by creating a binary column for each category (S1 Table). The present analysis received approval from the French Data Protection Authority (Commission Nationale de l’Informatique et des Libertés, authorization no 9191415; October 10, 2019) and was built in compliance with French and European regulations.

### Statistical analysis

#### Autoencoder

To adequately model a high-dimensional dataset, it is advantageous to perform dimensionality reduction prior to classification. Autoencoders are an efficient dimensionality reduction technique [25]. An autoencoder is a type of artificial neural network that learns a representation of the dataset while ignoring noise in the data; it can compress existing and missing information together, without the need for removing or imputing missing values. In this study, we used a classical autoencoder with one encoding function and one decoding function, and a binary cross entropy loss function.

To assess the difference in performance due to the use of an autoencoder, we performed two sets of analyses. In the first one, we performed dimensionality reduction of our data using an autoencoder and subsequently generated predictive models. In the other, the autoencoder was not used prior to data modeling.

#### Algorithms and performance measures

Predictive modeling is a branch of machine learning that uses data mining to predict results. In this study, we tested seven classification algorithms, and compared their performance in predicting discrete outcomes of interest. We used the Random Forest, Decision Tree, Naïve Bayesian, Linear Discriminant Analysis, Logistic Regression, Nearest Shrunken Centroids, and Neural Networks algorithms. We performed all analyses in Python programming language [Python Software Foundation, https://www.python.org] using *sklearn* [26] and *imblearn* [27] libraries with default parameter values.

We used 5-fold cross-validation, with 80% of the data being randomly selected for training and the remaining 20% for testing. We assessed model performance using accuracy, precision, recall, F1-score, and AUC score. We report averaged performance values over the five executions. Due to the imbalanced nature of our dataset, we selected our best-performing models based on the F1-score.

#### Predicted outcomes

Overall survival is widely accepted as the gold-standard primary endpoint. Because our goal was to develop a decision aid to assist clinicians in the choice of post-surgery treatment for elderly breast cancer patients, we focused on 5-year overall survival. Our approach was to generate outcome predictions for different courses of action using data from our cohort. The first outcome of interest was all-cause mortality at 5 years, where the objective was to predict whether a patient will die within five years from the date of surgery. The minority (positive) class consists of patients who have died within five years. The second outcome was the need for adjuvant treatment, where we consider that the choice of treatment was correct if the patient survived at least five years after surgery. In this case, we predicted whether a patient had any adjuvant treatment, given that they have survived. Hence, we are only interested in patients who have survived at least five years after surgery. The positive class consists of patients who did not undergo adjuvant treatment. The third predicted outcome was the need for adjuvant chemotherapy, where we assume that patients who underwent chemotherapy and survived at least five years after surgery were correctly treated, while the opposite holds for patients who had chemotherapy and did not survive at least five years. The positive class includes patients who underwent adjuvant chemotherapy. Finally, the fourth outcome was death after chemotherapy, where we aim to distinguish the patients who should not have undergone chemotherapy after surgery. The positive class is the group of patients who should not have undergone chemotherapy, i.e., patients who underwent chemotherapy after surgery and died within five years after surgery.

## Results

Patient demographics and characteristics were evaluated on the date of breast cancer diagnosis (Table 1). Median age was 75 years (range 69–96) with 233 (23.9%) patients aged 80 years or older. 160 (16.4%) patients were reported to have received chemotherapy, mainly in the adjuvant setting (141, 14.4%), with 18 (1.8%) in the neoadjuvant setting. Main comorbidities were diabetes (12.8% of patients), followed by coronary artery disease (10.3% of patients), and cardiac insufficiency (10.0% of patients).

**Table 1:** Patient characteristics.

Characteristic		N (%)
Primary breast tumor	Right	477 (48.9)
	Left	499 (51.1)
Age at diagnosis	69–74	471 (48.2)
	75–79	272 (27.9)
	80–84	156 (16.0)
	> = 85	77 (7.9)
SBR grade	1	176 (18.0)
	2	508 (52.0)
	3	274 (28.1)
	Missing	18 (1.9)
Hormone receptor status	ER-positive	813 (83.3)
	PR-positive	672 (68.9)
HER2 status	HER2-positive	72 (7.4)
Tumor size	T1	530 (54.3)
	T2	347 (35.6)
	T3	91 (9.3)
	Missing	8 (0.8)
Lymph node status	N0	560 (57.4)
	N1	269 (27.5)
	N2	71 (7.3)
	N3	43 (4.4)
	Missing	33 (3.4)
Type of surgery	Lumpectomy	505 (51.8)
	Mastectomy	493 (47.4)
	Missing	8 (0.8)
Endocrine therapy	Neoadjuvant	5 (0.5)
	Adjuvant	759 (77.8)
	Neoadjuvant and adjuvant	40 (4.1)
	Not performed	164 (16.8)
	Missing	8 (0.8)
Chemotherapy	Neoadjuvant	18 (1.8)
	Adjuvant	141 (14.5)
	Neoadjuvant and adjuvant	1 (0.1)
	Not performed	815 (83.5)
	Missing	1 (0.1)
Radiotherapy	Pre-surgery	7 (0.7)
	Post-surgery	454 (46.5)
	Post-chemotherapy	66 (6.8)
Second primary cancer	Yes	104 (10.7)
	No	814 (83.4)
	Missing	58 (5.9)
Vital status at last follow-up	Alive	610 (62.5)
	Dead	366 (37.5)

Algorithm performance measures are shown in Tables 2–5. Across all cases, logistic regression and/or linear discriminant analysis were the best performing models. We verified that the use of autoencoding generally improved model performance, with the exception of when we predicted the need for adjuvant treatment. To further assess the impact of the autoencoding step in model performance, we analyzed the information loss associated with the use of an autoencoder. Autoencoders generate a representation of a given input dataset using fewer dimensions that the original dataset. The encoding process of an autoencoder may lead to information loss. Information loss is the increase in entropy by transforming a dataset and is calculated by comparing the entropy of the dataset before and after a transformation.

**Table 2:** Results for prediction of death within five years.

	RF		NB		NSC		LDA		LR		NN		DT	
	–	AE	–	AE	–	AE	–	AE	–	AE	–	AE	–	AE
Accuracy	0.781	0.817	0.307	0.712	0.678	0.701	0.808	0.841	0.807	0.839	0.773	0.839	0.732	0.742
Precision	0.457	0.563	0.211	0.388	0.349	0.378	0.559	0.585	0.543	0.581	0.446	0.609	0.372	0.401
Recall	0.332	0.492	0.867	0.702	0.663	0.706	0.319	0.784	0.396	0.792	0.434	0.612	0.434	0.522
F1-score	0.380	0.521	0.338	0.498	0.456	0.490	0.401	**0.668**	0.454	**0.668**	0.436	0.607	0.397	0.450
AUC	0.615	0.696	0.515	0.708	0.672	0.703	0.627	0.820	0.655	0.822	0.647	0.755	0.622	0.660

**Table 3:** Results for prediction of the need for adjuvant treatment.

	RF		NB		NSC		LDA		LR		NN		DT	
	–	AE	–	AE	–	AE	–	AE	–	AE	–	AE	–	AE
Accuracy	0.983	0.981	0.983	0.983	0.943	0.913	0.986	0.959	0.990	0.988	0.989	0.988	0.990	0.969
Precision	0.901	0.852	0.937	0.774	0.404	0.304	0.782	0.490	0.880	0.815	0.885	0.839	0.890	0.592
Recall	0.633	0.623	0.609	0.822	0.916	0.949	0.911	0.982	0.879	0.907	0.830	0.874	0.877	0.639
F1-score	0.718	0.693	0.716	0.777	0.546	0.450	0.826	0.640	**0.869**	0.842	0.842	0.842	0.868	0.591
AUC	0.815	0.808	0.804	0.906	0.930	0.930	0.950	0.970	0.937	0.949	0.913	0.933	0.936	0.811

**Table 4:** Results for prediction of the need for adjuvant chemotherapy.

	RF		NB		NSC		LDA		LR		NN		DT	
	–	AE	–	AE	–	AE	–	AE	–	AE	–	AE	–	AE
Accuracy	0.865	0.876	0.307	0.791	0.733	0.782	0.902	0.922	0.903	0.927	0.889	0.929	0.847	0.828
Precision	0.608	0.629	0.175	0.416	0.331	0.403	0.753	0.694	0.710	0.724	0.651	0.778	0.519	0.470
Recall	0.435	0.551	0.907	0.759	0.658	0.767	0.577	0.912	0.665	0.884	0.661	0.778	0.590	0.568
F1-score	0.500	0.581	0.293	0.533	0.437	0.524	0.647	0.785	0.681	**0.792**	0.650	0.774	0.546	0.508
AUC	0.691	0.744	0.551	0.778	0.703	0.776	0.770	0.918	0.807	0.910	0.797	0.868	0.743	0.723

**Table 5:** Results for prediction of death after chemotherapy.

	RF		NB		NSC		LDA		LR		NN		DT	
	–	AE	–	AE	–	AE	–	AE	–	AE	–	AE	–	AE
Accuracy	0.621	0.669	0.565	0.679	0.657	0.677	0.706	0.831	0.703	0.824	0.669	0.774	0.650	0.629
Precision	0.665	0.713	0.736	0.703	0.674	0.701	0.753	0.847	0.736	0.837	0.685	0.791	0.672	0.652
Recall	0.577	0.632	0.273	0.682	0.684	0.682	0.666	0.831	0.687	0.830	0.699	0.782	0.663	0.650
F1-score	0.613	0.665	0.393	0.689	0.675	0.688	0.702	**0.837**	0.707	0.831	0.688	0.783	0.664	0.646
AUC	0.625	0.672	0.583	0.679	0.656	0.677	0.709	0.831	0.704	0.824	0.668	0.774	0.649	0.630

RF: Random Forest; NB: Naïve Bayesian; NSC: Nearest Shrunken Centroid; LDA: Linear Discriminant Analysis; LR: Logistic Regression; NN: Neural Network; DT: Decision Tree.

–: Model developed without the use of autoencoder; AE: Model developed with the use of autoencoder.

**Bold font** indicates best performing model based on F1-score value

In this study, we used an autoencoder to compress the input dataset before each predictive task. In other words, we generated a lower-dimensional dataset to predict death within five years, another lower-dimensional data to predict the need for adjuvant treatment, and so on. To quantify the information loss of the autoencoding step, we compared the entropy of the dataset before and after the transformation. We used the following equation to compute entropy, where p(x) denotes the probability of each possible outcome [28]:

Entropy=−∑p(x)log(p(x))

In Table 6, we report the average value and standard deviation of the information loss across 100 runs of the autoencoding step. In this analysis, a positive value indicates that the entropy of the compressed dataset is larger than that of the original dataset. Interestingly, we obtained overall best results using a logistic regression model without autoencoding to predict the need for adjuvant treatment (F1-score = 0.869).

**Table 6:** Information loss due to autoencoding.

Prediction task	Average information loss (standard deviation)
Predict death within five years	9.5 (5.5x10^-5^)
Predict the need for adjuvant treatment	9.2 (7.2x10^-5^)
Predict the need for adjuvant chemotherapy	9.2 (4.7x10^-5^)
Predict death after chemotherapy	7.7 (5.8x10^-5^)

To contribute with clinical insight, we performed a feature importance analysis to identify the most predictive features for each outcome of interest. Feature importance techniques assign a score to input features of a predictive model that indicates the relative importance of each feature when making a prediction. Inspecting the importance scores provides information about which features are the most and least important for the model when making a prediction.

Linear algorithms, such as linear discriminant analysis and logistic regression, fit a model where the predicted output is the weighted sum of the input values. These algorithms determine the set of coefficients to be used in the weighted sum in order to make a prediction. If we ensure that the input variables have the same scale or have been scaled prior to fitting the model, we can use the resulting coefficients directly as a type of feature importance score. In this work, we used library *sklearn* from Python to retrieve the property *coeff_* that contains the coefficients for each input variable of the linear discriminant analysis and logistic regression models. We then ranked all coefficients in decreasing order and retained the five highest ranked coefficients for each model.

Decision tree algorithms and ensembles of decision trees, such as random forest, offer importance scores based on the reduction in the criterion used to select split points, like Gini or entropy. After fitting the models using library *sklearn* in Python, we retrieved property *feature_importances_* that contains the relative importance score for each input feature of the decision tree and random forest models. We then ranked all scores in decreasing order and retained the five highest ranked scores for each model.

Results from this analysis are shown in Tables 7–10. The entries (numbers 1 thru 5) indicate the rank of each feature for each algorithm. For example, in Table 7 feature ‘Post-operative radiotherapy’ was ranked as the second most predictive feature in the Random Forest model and fifth most predictive feature in the Decision Tree model. Overall, the most predictive features across two or more models for any predictive task were ‘lymph node invasion (category 0–1)’, ‘adjuvant endocrine therapy’, and ‘post-chemotherapy radiotherapy’. Unsurprisingly, when predicting the need for adjuvant treatment, ‘adjuvant endocrine therapy’ was the most predictive feature across all four models. This result points to the importance of assessing the risk, or need, of adjuvant treatment in an elderly population. Classically, and as expected, the most predictive feature of 5-year overall survival is ‘Lymph node invasion–category 0–1’. Similarly, ’Lymph node invasion–category 0–1’ is the most predictive feature to predict the need for adjuvant chemotherapy. This implies that the extent of lymph node invasion can help to determine the likelihood of needing additional chemotherapy. In Table 10, ’Lymph node invasion–category 0–1’ is again a highly predictive feature when predicting death after chemotherapy. These results highlight the importance of lymph node invasion status as a key factor in patient outcomes.

**Table 7:** Most predictive features when predicting death within five years.

Feature	RF	LDA	LR	DT
Lymph node invasion–category 0–1	1		1	1
Histology–Invasive carcinoma		1		
Post-operative radiotherapy	2			5
Type of surgery–Lumpectomy		2		
GG sentinel performed–Yes			2	
Lymph node invasion–category 1–4				2
SBR grade–category 2	3			
Adjuvant endocrine therapy		3		4
IHC hormone receptor study performed			3	
Biggest tumor size–category 0–20 mm	4			3
Excision limit		4		
SBR grade–category 1			4	
Estrogen receptor level–category >80%	5			
SBR grade–NA		5		
Lymph node invasion–category 4–10			5	

**Table 8:** Most predictive features when predicting the need for adjuvant treatment.

Feature	RF	LDA	LR	DT
Adjuvant endocrine therapy	1	1	1	1
Endocrine therapy–not performed	2			
Site of radiotherapy–Right breast		2	2	4
Post-operative radiotherapy			5	2
Estrogen receptor level–category >80%	3			
Lymph node dissection–internal mammary		3		
Site of radiotherapy–Left breast			3	3
Excision limit	4			
Biggest tumor size–category 50–1000 mm		4		
SBR grade–category 3			4	
Type of surgery—Lumpectomy	5			
Site of radiotherapy–Left breast		5		
Monoclonal antibody therapy—Adjuvant				5

**Table 9:** Most predictive features when predicting the need for adjuvant chemotherapy.

Feature	RF	LDA	LR	DT
Lymph node invasion–category 0–1	1		2	1
Post-chemotherapy radiotherapy	2	1	1	2
Progesterone receptor level—Uninterpretable		2		
Estrogen receptor level–category >80%	3			4
Estrogen receptor level–Some marked cells		3		
Post-operative radiotherapy			3	5
Lymph node invasion–category 1–4				3
Progesterone receptor level–category >80%	4		5	
IHC hormone receptor study performed		4		
Monoclonal antibody therapy–Adjuvant			4	
Lymph node dissection–not performed	5			
Number of HER2 copied (ref. classification 2010)–category < = 6		5		

**Table 10:** Most predictive feature when predicting death after chemotherapy.

Feature	RF	LDA	LR	DT
Lymph node invasion–category 0–1	1		1	4
Adjuvant endocrine therapy–Other medication		1		
Post-operative radiotherapy	3			1
Adjuvant endocrine therapy–AA	2			5
Monoclonal antibody therapy–Not performed		2		
Lymph node invasion–category 4–10		5	2	3
Lymph node invasion–category 1–4	4		3	2
Muscle invasion		3		
Adjuvant endocrine therapy–Tamoxifen and AA		4		
Number of HER2 copies–category 5–8			4	
Performance status (WHO classification)–category 1	5			
Site of radiotherapy–Left breast			5	

RF: Random Forest; LDA: Linear Discriminant Analysis; LR: Logistic Regression; DT: Decision Tree.

## Discussion

Providing the most appropriate adjuvant treatment for elderly patients with early breast cancer represents a daily challenge for oncologists; this is partly due to the higher incidence of comorbid conditions in this frail population. Existing data provides limited strong evidence to support recommendations, and international guidelines do not provide tangible guidance for this group of patients. Our goal was to generate a decision aid for clinical decision-making on post-surgery treatment for elderly breast cancer patients. We identified four relevant scenarios that could assist clinicians in the choice of adjuvant therapy. First, we predicted whether a patient would die or not within five years after surgery. In this case, we assumed that we know which treatment a patient has received. Second, we predicted whether a patient would need any type of adjuvant treatment in order to survive at least five years. In a similar manner, we predicted whether a patient would need adjuvant chemotherapy in order to survive at least five years. We focused especially on chemotherapy because this decision is considered particularly complex and often feared by patients. Finally, we predicted whether the choice of chemotherapy was a good one, considering all patients who underwent this type of adjuvant therapy. In all our analyses, we assumed that a treatment was successful if the patient survived at least five years after surgery.

When predicting death within five years, ‘lymph node invasion (category 0–1)’ was the most predictive feature across three of the four models. Features ‘lymph node invasion (category 0–1)’ and ‘post-chemotherapy radiotherapy’ were most predictive of the need for adjuvant chemotherapy. Finally, ‘lymph node invasion (category 0–1)’ was again the most predictive feature of death after chemotherapy for two of the four models. These results are particularly interesting for oncologists because the aforementioned features relate only to tumor characteristics. In fact, the patient’s age or comorbidities were not ranked as highly significant prognostic factors in our models. This, in turn, suggests that the therapeutic management of elderly patients with localized breast cancer must be conducted similarly to what is done for other age groups. These findings are consistent with other retrospective studies, which report that the absence of adjuvant chemotherapy in this population may have an impact on the chances of overall survival [29–32].

We observed consistently better performance using logistic regression (LR) and linear discriminant analysis (LDA) models. LR outperformed all other models in two of the four predictive tasks. For the other two tasks, LR and LDA showed comparable predictive power. LR models suggest well-defined relationships that are typically highly interpretable. However, this interpretability may be hindered by the use of an autoencoder due to the trade-off between accuracy and interpretability. This occurs because autoencoding generates a compressed representation of the initial feature space, but it is usually infeasible to associate a clinical meaning with the compressed features. We observed that classification performance generally improved when autoencoder was used, except for when we predicted the need for adjuvant treatment. Interestingly, we obtained overall best results using LR without autoencoding to predict the need for adjuvant treatment (F1-score = 0.869).

We acknowledge limitations of our study, such as the imbalanced nature of our dataset, meaning that not all response classes included similar numbers of patients. Additional work could analyze the effect of class imbalance on model performance. Moreover, in this work we did not stratify patients based on, e.g., hormone receptor status or clinical staging prior to modeling. In the future, additional models could be generated for specific patient subgroups. Hence, external model validation could be performed for general and subgroup-specific models. We also acknowledge limitations in our dichotomous definition of outcome, where we consider that a choice is correct if the patient survives at least five years. Future work could consider a multi-factorial outcomes definition, by defining, e.g., a multi-objective function that incorporates different (possibly weighted) endpoints. Another limitation is that we did not predict outcomes such as recurrence. Finally, it would be relevant to evaluate our results in light of the oncogeriatric frailty scores. However, these geriatric evaluations are unfortunately still too infrequent in daily clinical practice to allow for such evaluation [33, 34].

## Supporting information

S1 Table Data collected for the study. Description of variables with their respective categories.
(DOCX)
