# Supplementary material for: Adjuvant therapeutic strategy decision support for an elderly population with localized breast cancer: A monocentric cohort retrospective study
Source: PLoS One. 2023 Aug 24;18(8):e0290566. doi: 10.1371/journal.pone.0290566 (PMC10449163; doi:10.1371/journal.pone.0290566)
Supplement: S1 Table — Description of variables with their respective categories. (DOCX) [file pone.0290566.s001.docx]

**S1 Table. Data collected for the study.** Description of variables with their respective categories.

| **Variable description** | **Categories** |
| --- | --- |
| Primary breast tumor | Right |
|  | Left |
| History of cancer | Yes |
|  | No |
| Performance status (WHO classification) | 0 |
|  | 1 |
|  | 2 |
|  | 3 |
|  | 4 |
| Body Mass Index | 0-18.5 |
|  | 18.5-25.0 |
|  | 25.0-30.0 |
|  | 30.0-90.0 |
| Type of surgery | Lumpectomy |
|  | Mastectomy |
| Lymph node dissection | Not performed |
|  | Axillary |
|  | Internal mammary |
|  | Both |
| Pre-chemo axillary lymph node dissection | Yes |
|  | No |
| Time of lymph node dissection | Primary |
|  | Secondary |
| GG sentinel performed | Yes |
|  | No |
| Pre-chemo GG sentinel lymph node dissection | Yes |
|  | No |
| Histology | Invasive carcinoma |
|  | Ductal cancer in situ |
|  | Lobular cancer in situ |
|  | Other |
| Tumor size (mm) | 0-20 |
|  | 20-50 |
|  | 50-1000 |
| Biggest tumor size (mm) | 0-20 |
|  | 20-50 |
|  | 50-1000 |
| Invasive lobular carcinoma | Yes |
|  | No |
| Invasive ductal carcinoma | Yes |
|  | No |
| SBR grade | 1 |
|  | 2 |
|  | 3 |
| Excision limit | Yes |
|  | No |
| Skin invasion | Yes |
|  | No |
| Nipple invasion | Yes |
|  | No |
| Pectoral muscle on surgical specimen | Yes |
|  | No |
| Muscle invasion | Yes |
|  | No |
| Lymph node invasion | 0-1 |
|  | 1-4 |
|  | 4-10 |
|  | 10-100 |
| IHC hormone receptor study performed | Yes |
|  | No |
| Estrogen receptor level | Uninterpretable |
|  | < 10% |
|  | 10-50% |
|  | 50-80% |
|  | > 80% |
|  | Some marked cells |
| Progesterone receptor level | Uninterpretable |
|  | < 10% |
|  | 10-50% |
|  | 50-80% |
|  | > 80% |
|  | Some marked cells |
| IHC HER2 study | Yes |
|  | No |
| HER2 expression level | 2 |
|  | 3 |
|  | Missing |
| FISH amplification performed | Yes |
|  | No |
| Number of HER2 copies | < 5 |
|  | 5-8 |
|  | >= 8 |
| Number of HER2 copies (ref. classification 2010) | <= 6 |
|  | > 6 |
| Ratio HER2/chromosome 17 centromeres | 0-2 |
|  | 2-100 |
| Complete histological response to neoadjuvant chemotherapy | Yes |
|  | No |
| Site of radiotherapy | Right |
|  | Left |
|  | Bilateral |
|  | Not performed |
| Post-operative radiotherapy | Yes |
|  | No |
| Perioperative radiotherapy | Yes |
|  | No |
| Post-chemotherapy radiotherapy | Yes |
|  | No |
| Endocrine therapy | Neoadjuvant |
|  | Adjuvant |
|  | Neoadjuvant and adjuvant |
|  | Not performed |
| Neoadjuvant endocrine therapy | Tamoxifen |
|  | Anti-aromatase (AA) |
| Adjuvant endocrine therapy | Tamoxifen |
|  | AA |
|  | Other |
|  | Tamoxifen and AA |
|  | AA and other |
| Monoclonal antibody therapy | Neoadjuvant |
|  | Adjuvant |
|  | Neoadjuvant and adjuvant |
|  | Not performed |
| Chemotherapy | Neoadjuvant |
|  | Adjuvant |
|  | Neoadjuvant and adjuvant |
|  | Not performed |
| G-8 score | 0-14 |
|  | 14-100 |
| Number of yearly hospitalizations | Value |
| Diabetes | Yes |
|  | No |
| Cardiac insufficiency | Yes |
|  | No |
| Coronary artery disease | Yes |
|  | No |
| Chronic obstructive pulmonary disease | Yes |
|  | No |
| Hemoglobin (g/dL) | 0-8 |
|  | 8-10 |
|  | 10-100 |
| Creatinine (ml/mi) | 0-15 |
|  | 15-30 |
|  | 30-60 |
|  | 60-500 |
| Lymphocytes (g/L) | 0-0.2 |
|  | 0.2-0.5 |
|  | 0.5-0.8 |
|  | 0.8-1000 |
| Albuminemia | 0-20 |
|  | 20-30 |
|  | 30-100 |
| Polypharmacy (> or = 5 medications a day) | Yes |
|  | No |
